# Impact of Angiosperm Tree-Derived Allelochemicals on Physiological Responses of Acceptor Plants—A Systematic Review

**DOI:** 10.3390/ijms27146188

**Published:** 2026-07-10

**Authors:** Maja Paterska, Konrad Osowski

**Affiliations:** 1 Department of Plant Physiology, Poznan University of Life Sciences, Wołyńska 35, 60-637 Poznan, Poland; 2 Faculty of Agriculture, Horticulture and Biotechnology, Poznań University of Life Sciences, Wojska Polskiego 28, 60-637 Poznan, Poland; 98015@student.up.poznan.pl

**Keywords:** allelopathy, allelochemicals, angiosperm trees, acceptor plants, phytotoxicity, oxidative stress, photosynthesis, plant physiology, secondary metabolites, agroforestry

## Abstract

Allelopathy—the release of biologically active secondary metabolites by plants—is a key mechanism regulating plant community dynamics in forest and agro-forestry ecosystems, yet the physiological basis of allelochemical effects of angiosperm trees on acceptor plants remains insufficiently understood. This systematic review synthesises knowledge on the physiological effects of angiosperm tree allelochemicals, focusing on photosynthesis, respiration, water relations, mineral nutrition, and growth and development. Following PRISMA 2020 guidelines, five databases (Web of Science, ScienceDirect, SpringerLink, Wiley Online Library, and Scopus) were searched between October 2025 and June 2026 for English-language peer-reviewed articles on allelopathic effects on plant physiological processes; risk of bias was assessed via methodological transparency, and, given design heterogeneity, a qualitative narrative synthesis was performed. Of 7748 records identified, 99 met the eligibility criteria. Allelochemicals, including phenolic acids, flavonoids, naphthoquinones, terpenoids, alkaloids, and coumarins, were associated with disrupted photosynthetic efficiency, impaired mitochondrial electron transport, altered stomatal functioning, reduced nutrient uptake, and suppressed cell division, with oxidative stress—often linked to reactive oxygen species accumulation—recurring as a shared mechanism; effects were concentration-dependent, with synergistic interactions noted between allelochemicals. These findings advance mechanistic understanding of angiosperm tree allelopathy and highlight its relevance for sustainable agriculture and biological weed management.

## 1. Introduction

Allelopathy, defined as the direct or indirect chemical interaction between one plant organism and another through the release of secondary metabolites—termed allelochemicals—into the environment, constitutes one of the key mechanisms regulating the structure and dynamics of plant communities [1,2]. This phenomenon, known since antiquity, when Theophrastus described the detrimental effects of chickpea on neighbouring plants, whilst Pliny the Elder noted the phytotoxic activity of walnut (*Juglans regia* L.) on vegetation growing in its vicinity, did not acquire a rigorous scientific foundation until the twentieth century, when Hans Molisch coined the term itself and was the first to systematically characterise chemical interactions between plants (Molisch, 1937, cited in [1]). Of fundamental importance to the development of this discipline were the works of Rice [1], who compiled and systematised the existing knowledge on plant-chemical interactions, and the subsequent studies of Inderjit [3] concerning the role of soil as a medium for the transformation and transport of allelochemicals. The contemporary definition of allelopathy, proposed by Torres and the International Allelopathy Society (IAS) in 1996, broadens this concept to encompass all chemical interactions between plants, fungi, bacteria, and algae, including both inhibitory and stimulatory effects—a formulation that reflects the growing recognition of the complexity of chemical interactions in ecosystems. As allelopathy remains an emerging field of inquiry, it is natural that its definition continues to evolve [4,5,6].

A key element distinguishing allelopathy from competition lies in the mechanism of interaction: whereas competition involves the depletion of a shared pool of environmental resources, allelopathy is based on the active release of chemical compounds that modify the physiology of target organisms independently of resource availability [2,7]. In ecological practice, both mechanisms frequently co-occur and mutually reinforce one another, which represents one of the principal methodological challenges in allelopathy research and necessitates the use of precisely designed experimental systems capable of discriminating between them. This issue is of particular relevance in the context of field studies, where the conditions of natural plant communities preclude full control of environmental variables [2].

Among all groups of vascular plants, trees occupy a particularly prominent position in allelopathy research. Their biological characteristics—considerable above- and below-ground tissue biomass, an extensive root system, longevity, and the capacity for intensive production and release of secondary metabolites throughout the entire growing season—render them exceptionally effective allelochemical donors in forest and agroforestry ecosystems. In this context, an important distinction must be drawn between the allelopathy of angiosperm trees and that of conifers, which, despite superficial similarities, differ fundamentally in the chemical profile of their allelochemicals, the pathways of their release, and the mechanisms by which they act upon acceptor plants [8,9].

The allelopathy of conifers is based primarily on the production and emission of monoterpenes and sesquiterpenes, which constitute the principal components of essential oils present in resin, needles, and bark [10]. Among the best-characterised allelochemicals of coniferous trees are α-pinene, β-pinene, camphor, 1,8-cineole, and terpinen-4-ol, emitted intensively by species of the genera *Pinus*, *Abies*, *Picea*, and *Thuja*. Conifer litter is characterised by a high content of resins, condensed tannins, and polyphenols of proanthocyanidin structure, which are released slowly during decomposition and acidify the soil, creating a specific chemical environment with inhibitory properties towards a broad spectrum of acceptor plants [11]. Coniferous litter decomposes more slowly than angiosperm tree litter, owing to its higher lignin content and lower C:N ratio, resulting in the gradual release of allelochemicals over an extended period and sustaining chronic, low-level chemical stress in the soil beneath the tree canopy [12].

The allelopathy of angiosperm trees exhibits a number of features that distinguish it from that of conifers, arising both from the distinct chemical profile of the metabolites produced and from the specific nature of the seasonal biological cycle. First, angiosperm trees of the temperate zone produce a chemically far more diverse array of allelochemicals, encompassing—alongside terpenoids—phenolic acids, flavonoids, naphthoquinones, alkaloids, and coumarins, which translates into a broader spectrum of phytotoxic mechanisms and a greater diversity of acceptor organisms. Second, the annual leaf fall of angiosperm trees generates and releases allelochemicals of a markedly seasonal character, distinctly different from the continuous, slow release that characterises decomposing coniferous litter, implying that the intensity of allelopathic stress beneath angiosperm trees exhibits a pronounced annual rhythmicity, with a peak during the autumn–winter period following leaf abscission [13]. Third, angiosperm trees of the temperate and tropical zones frequently produce highly specific and structurally unique allelochemicals—such as juglone in *Juglans* spp. or mimosine in *Leucaena leucocephala* (Lam.) de Wit—with exceptionally high phytotoxic potential and precise molecular targets, a phenomenon far less commonly observed in conifers. Fourth, soils beneath angiosperm trees are generally characterised by higher pH and greater microbial activity than soils under coniferous stands, which promotes more rapid transformation of phenolic allelochemicals and may lead both to their detoxification and to the generation of forms with enhanced phytotoxicity [14]. Given these fundamental differences, the present review focuses specifically on allelochemicals derived from angiosperm trees, while a comparative analysis with coniferous and other evergreen species lies beyond its intended scope, and may constitute a valuable direction for future research.

The study of angiosperm tree allelopathy is justified from several mutually reinforcing scientific, ecological, and applied perspectives. From an ecological standpoint, allelopathy constitutes an important yet still insufficiently understood mechanism regulating the species composition of the forest understorey, succession dynamics, and mineral nutrient cycling in forest ecosystems. Understanding the chemical basis of these interactions is of critical importance for the interpretation of biodiversity patterns in angiosperm forests, where the dominance of individual tree species frequently correlates with impoverishment of the ground flora and shrub layer in their immediate vicinity [15].

From the perspective of ecological threats, a particularly urgent concern is the allelopathy of invasive tree species such as *Ailanthus altissima* (Mill.) Swingle, *Rhus typhina* L., and *Acer negundo* L., which—through the intensive release of allelochemicals—may permanently modify the chemical and biological properties of the soil, eliminate native herbaceous and shrub vegetation, and impede the natural regeneration of native forest communities [16,17]. At the global scale, biological invasions by woody plant species generate enormous ecological and economic losses, and understanding the allelopathic mechanisms underpinning these processes is a prerequisite for the development of effective invasive-species management strategies [18]. An equally significant concern is the phenomenon of autotoxicity observed in monoculture plantations of angiosperm tree species—particularly in plantations of *Eucalyptus* spp., *Populus* spp., and *Juglans* spp.—where the accumulation of allelochemicals in the soil leads to inhibition of natural regeneration and a reduction in the productivity of successive tree generations [19,20].

From the perspective of opportunities and applications, the allelopathy of angiosperm trees represents a promising source of natural bioherbicides and biopesticides, the development of which aligns with the global imperative to reduce the use of synthetic plant-protection products in agriculture and forestry [8,21]. Identified angiosperm tree allelochemicals—such as juglone, mimosine, and hydroxycinnamic acids—exhibit selective inhibitory activity against weeds whilst maintaining lower toxicity towards crop plants, rendering them candidate compounds for applications in organic and precision agriculture [22]. Furthermore, agroforestry systems based on angiosperm tree species with a controlled allelopathic potential may be deliberately designed to achieve biological weed suppression, reduction of soilborne pathogen pressure, and manipulation of the species composition of associated crop vegetation [14]. Finally, elucidating the molecular mechanisms by which angiosperm tree allelochemicals act upon the photosynthetic apparatus, mineral and water relations, and the respiratory metabolism of acceptor plants opens the prospect of utilising these compounds as research tools in plant physiology and as templates for the rational design of novel classes of herbicides with precise molecular targets [23].

A significant and hitherto insufficiently recognised methodological limitation of contemporary allelopathy research is its restricted analytical scope. A substantial proportion of the available publications conclude at the bioassay stage—demonstrating inhibition of germination, reduction in root length, or suppression of seedling growth—without attempting to elucidate the physiological and biochemical basis of the observed effects. Whilst this approach is valuable from the perspective of documenting the allelopathic phenomenon, it does not permit the identification of specific molecular targets of allelochemicals, the mechanisms underlying metabolic disruption, or the signalling pathways activated in response to allelopathic stress. As a consequence, knowledge of angiosperm tree allelopathy remains largely descriptive and phenomenological, rather than mechanistic and predictive. There is a conspicuous absence of synthetic reviews integrating bioassay data with knowledge of the physiological and biochemical underpinnings of phytotoxicity—a critical gap that impedes both a deeper understanding of the ecological role of allelopathy and the practical exploitation of angiosperm tree allelochemicals in plant protection and sustainable agriculture.

Several reviews have already contributed significantly to the understanding of allelopathy by addressing the ecological importance of allelopathic interactions and the diversity, classification, and chemical characteristics of allelochemicals, as well as the biological activity and phytotoxic potential of major groups of allelopathic compounds. Other studies have focused on specific plant species, selected allelochemical classes, or general mechanisms associated with phytotoxicity. However, existing reviews have generally considered allelopathy from a broad perspective, and have rarely focused specifically on the physiological consequences of allelochemicals released by angiosperm trees in acceptor plants. In particular, the relationship between the chemical nature of tree-derived allelochemicals and their effects on key physiological processes, including photosynthesis, respiration, water relations, mineral nutrition, and growth regulation, remains insufficiently integrated.

It should be emphasised that allelopathy research is characterised by considerable diversity in experimental approaches, including studies based on plant extracts, purified compounds, and plant-derived materials, as well as different growth systems and environmental conditions. This variability may affect the availability of allelochemicals, the intensity of their biological activity, and the range of plant responses observed. Therefore, interpretation of allelopathic effects requires recognition that outcomes reported in individual studies reflect not only the chemical properties of the investigated compounds, but also the characteristics of the experimental system applied. The present review focuses primarily on identifying recurring physiological and biochemical changes occurring in acceptor plants and on elucidating the mechanisms underlying these responses.

Unlike previous reviews, the present systematic review adopts a physiology-oriented perspective that integrates the chemical diversity of angiosperm tree-derived allelochemicals with their mechanisms of action in acceptor plants. Rather than summarising the occurrence, classification, or phytotoxic potential of individual compounds, this review synthesises current evidence linking chemical classes, environmental release pathways, and physiological responses across five major domains of plant functioning. By integrating findings from molecular, biochemical, physiological, and ecological studies, this review provides a comprehensive mechanistic framework for understanding how angiosperm tree-derived allelochemicals influence plant performance, and identifies key directions for future research.

A central element in the physiological response of acceptor plants to allelochemicals is oxidative stress, which has been increasingly recognised as one of the key mechanisms underlying phytotoxicity. However, the role of oxidative stress in allelopathic interactions remains complex and should not be interpreted exclusively as either a primary cause or a secondary consequence of physiological disruption. Redox-active allelochemicals, particularly naphthoquinones such as juglone and selected phenolic compounds, may directly induce reactive oxygen species (ROS) accumulation through interference with mitochondrial and chloroplast electron transport processes and disruption of cellular redox homeostasis [24,25,26,27]. Conversely, ROS generation may also occur as a consequence of preceding disturbances in photosynthesis, respiration, membrane stability, and cellular metabolism [26,28,29]. Therefore, oxidative stress may function simultaneously as an initiating mechanism, a consequence of metabolic dysfunction, and an amplifying component integrating multiple physiological responses during allelochemical exposure [16,27].

**Figure 1:**
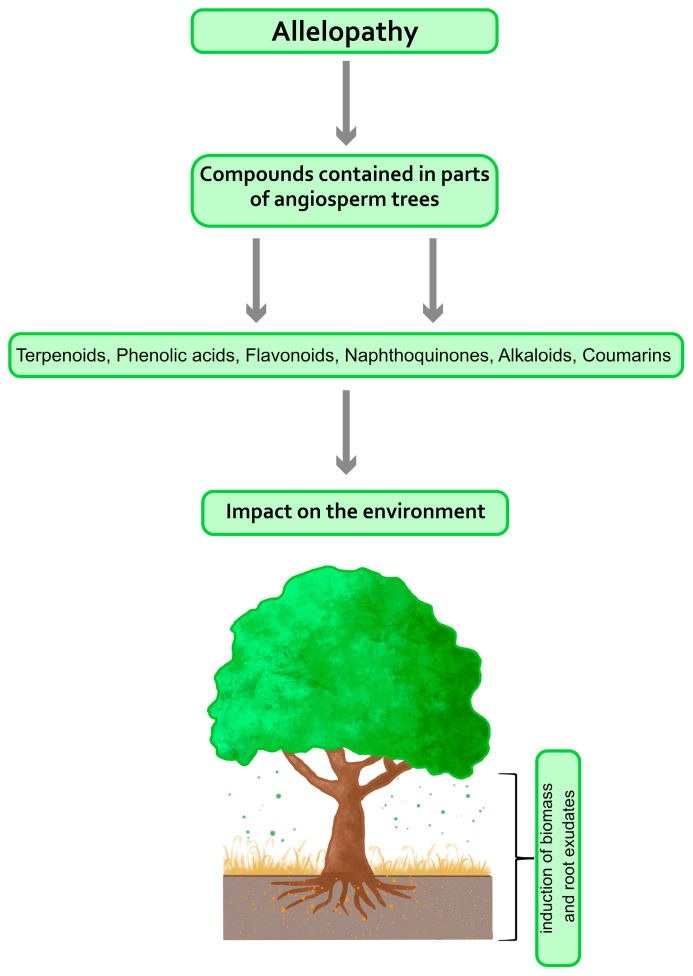
Angiosperm trees release structurally diverse allelochemicals via multiple environmental pathways, exerting multi-level and synergistic phytotoxic effects on the photosynthesis, respiration, water relations, mineral nutrition, and growth of acceptor plants, with oxidative stress emerging as the central integrating mechanism.

The present systematic review aims to address this identified research gap through a synthetic and critical appraisal of the current state of knowledge on the effects of allelochemicals emitted by angiosperm trees on acceptor plant physiology. Specifically, this review addresses the following question: What are the physiological effects of allelochemicals released by angiosperm tree species on acceptor plants, and what mechanisms underlie these effects? The thematic scope of the review encompasses interactions affecting five key domains of acceptor plant functioning: photosynthesis, respiration, water relations, mineral nutrition, and growth and development, examined in the context of the principal chemical classes of allelochemicals and the diverse pathways of their environmental release. Particular emphasis is placed on the multi-level and frequently synergistic nature of these interactions, their ecological significance and applied implications, and the imperative to advance allelopathy research beyond a purely phenomenological framework towards a mechanistic understanding of the physiological basis of phytotoxicity (Figure 1).

## 2. Materials and Methods

### 2.1. Study Design

A systematic review of the scientific literature was conducted in accordance with the Preferred Reporting Items for Systematic Reviews and Meta-Analyses (PRISMA 2020) guidelines [30]. This review was not registered in PROSPERO or any other prospective register; however, eligibility criteria and the search strategy were defined prior to data collection.

All illustrations and graphical elements presented in this manuscript were prepared using the freely available versions of the graphic design software packages Krita 5.3.2.1 and Affinity 3.2.2. No licensed or commercially licensed software was used for the creation of the figures.

### 2.2. Search Strategy

Independent searches were performed across 5 electronic databases: Web of Science (*n* = 598), ScienceDirect (*n* = 2056), SpringerLink (*n* = 2448), Wiley Online Library (*n* = 1923), and Scopus (*n* = 723). Searches were conducted between October 2025 and June 2026. The search strategy was based on Boolean combinations of keywords encompassing genus names of angiosperm tree species and terms related to physiological processes under investigation, including allelopathy, photosynthesis, water relations, mineral nutrition, growth, development, and respiration. No date restrictions were applied. The search was limited to English-language peer-reviewed journal articles.

### 2.3. Eligibility Criteria

Inclusion and exclusion criteria were defined a priori. Studies were included if they met all of the following criteria:

- Original research articles.
- Published in English in peer-reviewed scientific journals.
- Reporting allelopathic effects of angiosperm tree species on plant physiological processes.

Studies were excluded if they met any of the following criteria:

- Books, book chapters, conference abstracts, or review articles.
- Studies concerning coniferous tree species.
- Publications reporting bioassay results only.
- Studies focused on medical applications of allelochemicals.
- Studies limited to chemical characterization of allelochemicals without assessment of physiological effects.
- No access to full text.

### 2.4. Study Selection

**Figure 2:**
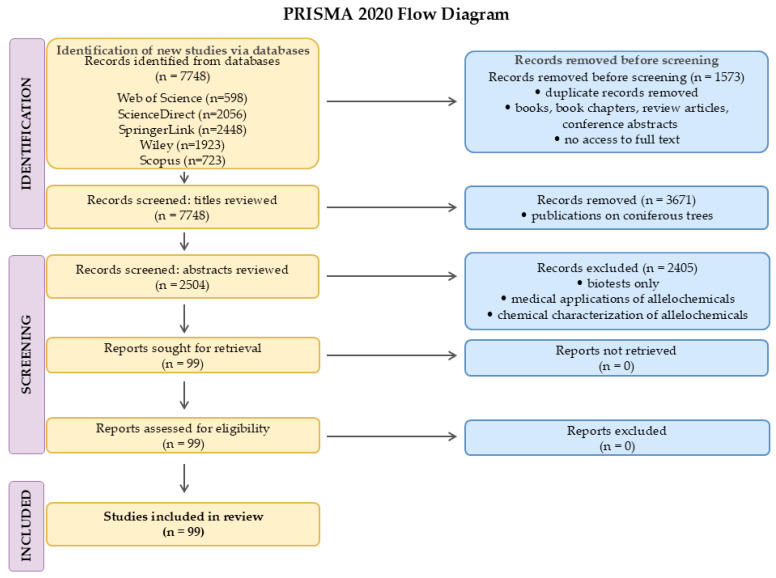
PRISMA 2020 flow diagram.

The study selection process was conducted in three sequential stages, consistent with the PRISMA 2020 framework, and is illustrated in the PRISMA 2020 flow diagram (Figure 2).

Stage 1—Identification: database searches yielded a total of 7748 records. No additional records were identified through searches of grey literature, citation tracking, or other sources. Prior to screening, 1573 records were removed due to duplication or ineligibility (books, book chapters, conference abstracts, review articles, and inaccessible full texts).

Stage 2—Screening: title screening of the remaining 7748 records resulted in the exclusion of 3671 records pertaining to coniferous tree species, which fell outside the thematic scope of the review. The remaining 2504 records proceeded to abstract screening, at which stage a further 2405 records were excluded on the following grounds: bioassay-only studies (without assessment of physiological parameters), medical applications of allelochemicals, and chemical characterization of allelochemicals without physiological context.

Stage 3—Eligibility assessment and inclusion: the remaining 99 records were subjected to full-text assessment against the predefined eligibility criteria. All 99 records satisfied the inclusion criteria, and were incorporated into the final qualitative synthesis.

### 2.5. Data Extraction and Synthesis

Data were extracted from the included studies in a standardized manner. The following information was recorded for each publication: author(s), year of publication, donor and recipient plant species, type of allelopathic material, physiological parameters assessed, experimental conditions, and key findings. Due to the heterogeneity of experimental designs and reported outcomes across the included studies, a quantitative synthesis (meta-analysis) was not performed. Instead, a qualitative narrative synthesis was conducted, organized thematically according to the physiological processes investigated. Upon completion of the selection process, a final corpus of 99 publications was included in the review, constituting the basis for the qualitative synthesis of data on the allelopathic effects on plant physiological processes.

## 3. Results

### 3.1. Chemical Classes of Allelochemicals Emitted by Angiosperm Trees

**Figure 3:**
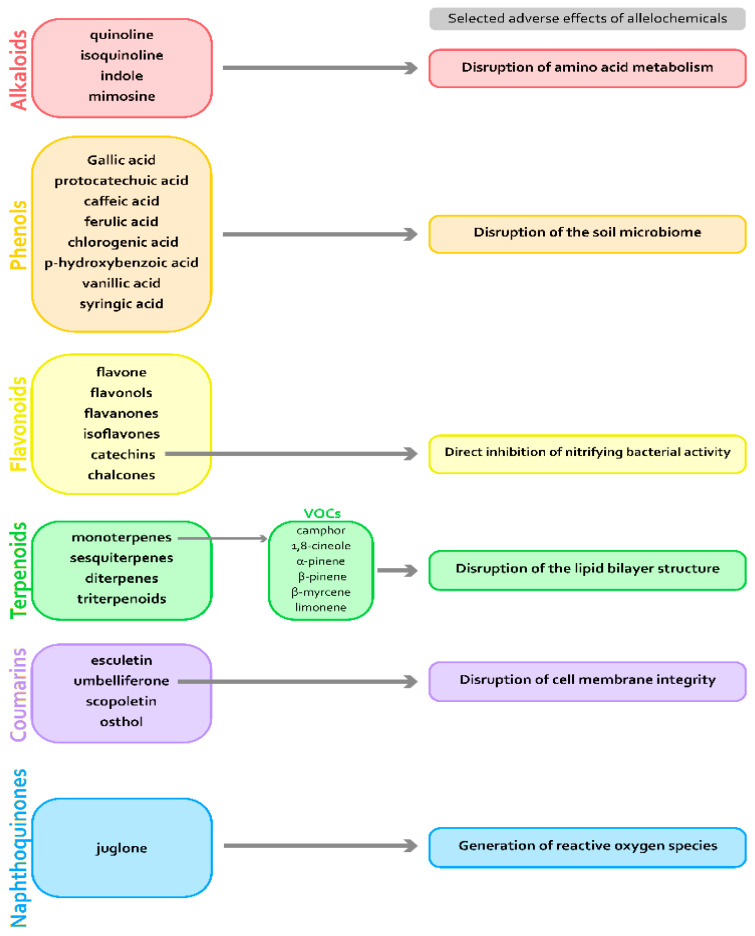
Overview of major chemical classes of allelochemicals and their reported adverse effects on target physiological and biochemical processes in acceptor plants, based on evidence synthesised in this systematic review. Allelochemicals can be grouped into several major chemical classes, including alkaloids, phenolics, flavonoids, terpenoids (including volatile organic compounds), coumarins, and naphthoquinones. Each class comprises representative compounds with distinct modes of action. Alkaloids (e.g., quinoline, isoquinoline, indole, mimosine) are associated with disruption of amino acid metabolism. Phenolic compounds (e.g., gallic, caffeic, ferulic, chlorogenic, vanillic, syringic acids) affect soil–plant interactions through disruption of the soil microbiome. Flavonoids (e.g., flavones, flavonols, flavanones, isoflavones, catechins, chalcones) have been linked to inhibition of nitrifying bacterial activity. Terpenoids, including mono-, sesqui-, di-, and triterpenes, as well as volatile organic compounds (e.g., camphor, 1,8-cineole, α- and β-pinene, β-myrcene, limonene), disrupt lipid bilayer structure and membrane-associated processes. Coumarins (e.g., esculetin, umbelliferone, scopoletin, osthol) impair cell membrane integrity, while naphthoquinones (e.g., juglone) are associated with the generation of reactive oxygen species (ROS). Collectively, these compound classes contribute to multiple, overlapping mechanisms of phytotoxicity affecting plant physiological and biochemical homeostasis.

Angiosperm trees produce a broad array of secondary metabolites with documented allelopathic potential, belonging to several distinct chemical classes (Figure 3). These compounds are released into the environment via multiple pathways, including leaching from above-ground tissues by precipitation, decomposition of leaf and root litter, active and passive root exudation, and emission of volatile organic compounds (VOCs) into the atmosphere [31]. The chemical composition and emission intensity of allelochemicals are determined by tree species, tissue age and type, abiotic conditions, and interactions with the soil microbiome, which may both degrade and biologically activate specific compound classes through enzymatic transformation of their chemical structures [12,14,25]. Despite this variability, the most extensively documented classes of allelopathically active compounds in angiosperm trees include simple phenols and phenolic acids, flavonoids, naphthoquinones, terpenoids, alkaloids, coumarins, and VOCs [32,33]. Each of these classes is characterised by a distinct physicochemical profile, release pathways, and mechanisms of action on acceptor plants, which determines their ecological role in shaping plant communities [33,34].

Simple phenols and phenolic acids constitute the most extensively described and best-documented class of allelochemicals in angiosperm trees, occurring ubiquitously in leaves, roots, bark, and decomposing leaf and root litter. Among the best-characterised representatives of this group are gallic acid, protocatechuic acid, caffeic acid, ferulic acid, chlorogenic acid, p-hydroxybenzoic acid, vanillic acid, and syringic acid [35,36]. These compounds are characterised by the presence of an aromatic ring bearing one or more hydroxyl substituents, which underlies their capacity to form hydrogen bonds with enzymatic proteins and to form chelate metal ions and interact with the phospholipids of biological membranes [37,38]. In the soil, phenolic acids accumulate in the rhizosphere of trees, from which they are taken up by the roots of acceptor plants or interact directly with the soil microbiome responsible for nitrogen and carbon cycling. Their bioavailability in the soil is dependent upon pH, organic matter content, mineral composition, and microbial activity; under anaerobic conditions, they may undergo conversion to forms of enhanced phytotoxicity [39].

Flavonoids constitute a structurally diverse group of polyphenols based on the diphenylpropane (C6–C3–C6) skeleton, encompassing flavones, flavonols, flavanones, isoflavones, chalcones, and catechins, which occur ubiquitously in the leaf tissues of temperate angiosperm trees [40]. The degree of hydroxylation, methylation, and glycosylation of the aromatic rings determines their physicochemical properties, including water solubility, persistence in the soil environment, and selectivity of interaction with molecular targets in the cells of acceptor plants [16,40]. Flavonoids are released into the soil both via root exudates and during the decomposition of leaf litter; their biological activity in the soil may be modulated by adsorption onto mineral particles and humic substances, as well as by photodegradation in the surface soil layers [41].

Naphthoquinones constitute a class of quinone secondary metabolites with exceptionally high phytotoxic potential, the most representative member of which among angiosperm trees is juglone (5-hydroxy-1,4-naphthoquinone), produced by species of the genus *Juglans*. Juglone is synthesised in the leaves, roots, and fruit husks in the form of the inactive glucoside hydrojuglone, which—following release into the soil via tissue leaching and litter decomposition—undergoes enzymatic and chemical oxidation to the toxic quinone form. The high phytotoxic reactivity of juglone is attributable to its redox properties —specifically, its capacity to generate reactive oxygen species (ROS) through cyclic redox reactions with cellular enzymatic systems, as well as its direct reactivity with the thiol groups of enzymatic proteins [24,41]. Under conditions of high soil organic-matter content, juglone may form stable complexes with humic substances, thereby extending its biological activity over time [25].

Terpenoids—encompassing mono-, sesqui-, di-, and triterpenoids—represent the numerically largest class of plant secondary metabolites, and their allelopathic activity in angiosperm trees is particularly well documented for species of the genera *Cinnamomum*, *Eucalyptus*, and *Ailanthus* [42]. Monoterpenes such as camphor, 1,8-cineole, α-pinene, β-pinene, β-myrcene, and limonene constitute the principal components of VOCs emitted from the leaves and bark of angiosperm trees; their phytotoxicity arises from their high lipophilicity, which enables unrestricted penetration of cell membranes, disruption of the lipid bilayer structure, inhibition of mitochondrial enzymes, and interference with hormonal signalling pathways in acceptor plants [41]. Sesquiterpenes, present in both the volatile fraction and root exudates, exhibit particular activity against the photosynthetic apparatus and cell division processes in root meristems. Monoterpenes and sesquiterpenes are released into the environment primarily via the volatile pathway from secretory glands on the leaf surface; however, following deposition onto the soil, they may accumulate in its surface layers and exert effects on germinating seeds and seedlings of acceptor plants [40].

Alkaloids constitute a class of nitrogenous secondary metabolites with complex heterocyclic structures, whose allelopathic activity in angiosperm trees is particularly well documented for mimosine—a non-protein amino acid produced by *Leucaena leucocephala*—as well as for quinoline, isoquinoline, and indole alkaloids present in species of the genera *Ailanthus*, *Juglans*, and *Acer* [43,44,45,46]. Mimosine, a structural analogue of tyrosine and phenylalanine, exerts multidirectional phytotoxic effects through competitive inhibition of pyridoxal phosphate-dependent enzymes, suppression of nitrate reductase activity in the leaf tissues of acceptor plants, disruption of amino acid metabolism, and formation of stable chelate complexes with transition metal ions. Alkaloids are released into the soil environment primarily through leaching from leaves and root litter and through biomass decomposition; their persistence in the soil is variable, and dependent upon chemical structure, the sorption properties of the substrate, and microbial activity [47].

Coumarins constitute a class of cinnamic acid-derived lactones present in the tissues of numerous angiosperm trees, including *Ailanthus altissima* and species of the genus *Acer* [48]. Allelopathically active representatives of this class include esculetin, umbelliferone, scopoletin, and osthole, which interfere with the auxin metabolism of acceptor plants through inhibition of indole-3-acetic acid oxidase activity, disrupt cell membrane integrity, and exhibit inhibitory activity against seed germination and root elongation. Coumarins are released into the environment both via root exudates and through leaching from above-ground tissues; in the soil, they may undergo photochemical activation to forms of enhanced phytotoxicity [41].

The allelochemicals emitted by angiosperm trees constitute a chemically diverse array of secondary metabolites whose synergistic or additive effects on acceptor plants cannot readily be reduced to the action of any single compound [25,33]. The complexity of the allelochemical mixtures released by a single tree species, their transformation in the soil environment, and their modification by the rhizospheric microbiome collectively imply that understanding the mechanisms of angiosperm tree allelopathy requires a multi-level approach that integrates both environmental chemistry and the physiology of acceptor plants [33].

### 3.2. Effects of Allelochemicals Emitted by Angiosperm Trees on Photosynthesis

**Figure 4:**
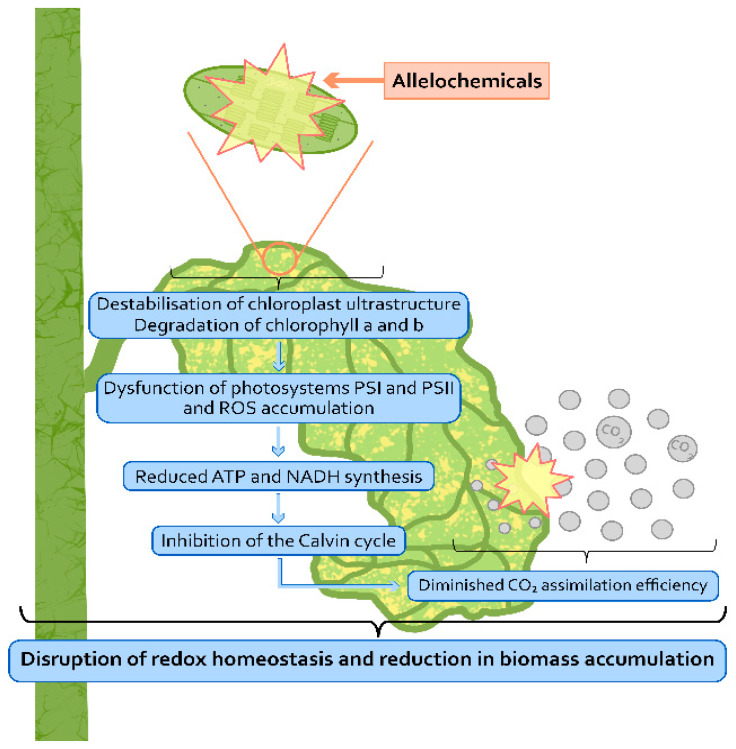
Conceptual summary of the reported effects of allelochemicals on the photosynthetic apparatus and plant growth, based on evidence synthesised in this systematic review. Following exposure to allelochemicals, chloroplast ultrastructure becomes destabilised, accompanied by the degradation of chlorophyll a and b. These alterations impair the function of photosystems I (PSI) and II (PSII), resulting in the accumulation of reactive oxygen species (ROS) and disruption of photosynthetic electron transport. The consequent reduction in ATP and NADPH production limits Calvin cycle activity and decreases CO_2_ assimilation efficiency. Collectively, these physiological and biochemical changes disturb cellular redox homeostasis, leading to impaired photosynthetic performance and, ultimately, educed biomass accumulation and plant growth. The figure presents a conceptual model integrating the principal mechanisms consistently reported across the studies included in this systematic review.

Allelochemicals emitted by angiosperm trees significantly modify the course of photosynthesis in acceptor plants (Figure 4). The principal classes of allelochemicals responsible for these effects include simple phenols, phenolic acids, flavonoids, and naphthoquinone metabolites [28,49]. Their action gives rise to multi-level disruptions of photosynthetic apparatus function, encompassing degradation and inhibition of photosynthetic pigment biosynthesis, dysfunction of photosystems PSI and PSII, accompanied by impairment of electron transport, restriction of gas exchange, and induction of oxidative stress, resulting in destabilisation of chloroplast ultrastructure [26,27,50]. These processes are closely interrelated, and their severity is dependent upon the concentration and chemical composition of the allelochemicals present [28,51].

One of the most frequently observed consequences of allelochemical action by angiosperm trees is a reduction in the contents of chlorophyll a, chlorophyll b, and carotenoids, which directly diminishes the capacity for light-nergy absorption and the efficiency of net photosynthesis [52,53]. The effect in question was demonstrated in a pot experiment, in which freshly fallen leaf litter of *Cinnamomum septentrionale* Hand.-Mazz. was incorporated directly into the soil at three application rates—25, 50, and 75 g per pot (10 kg soil)—and allowed to decompose for 50 days, after which the physiological parameters of the recipient plants *Zea mays* L., *Cucumis sativus* L., and *Vigna unguiculata* (L.) Walp. were assessed. With increasing litter dose, chlorophyll (a + b) and carotenoid contents declined significantly in all three species, with the strongest inhibition observed in maize and cucumber at the highest dose and in cowpea already at the intermediate dose, reaching reductions of up to 45.8%, 41.3%, and 33.3% for total chlorophyll and 35.3%, 32.1%, and 35.2% for carotenoids, respectively. In parallel, *Z. mays* and *V. unguiculata* showed a significant decline in net photosynthetic rate, stomatal conductance, and transpiration rate with increasing litter dose, while intercellular CO_2_ concentration increased, indicating a non-stomatal limitation of photosynthesis; in *C. sativus*, net photosynthetic rate and the associated gas-exchange parameters were generally reduced relative to the control, with the decline in net photosynthetic rate reaching 33.4% and 33.7% at the lowest and intermediate doses, respectively. The accompanying inhibition of vegetative growth included a significant reduction in biomass and leaf area in all three species, reaching up to 28.5% and 25.2% in maize, 26.6% and 26.5% in cucumber, and, in cowpea—which displayed a non-linear, dose-dependent response peaking at the intermediate dose—up to 29.1% and 14.4% [52]. Leaf extracts of *Eucalyptus camaldulensis* Dehnh. acting upon *Vigna radiata* (L.) R.Wilczek seedlings were shown to induce a significant, concentration-dependent reduction in chloroplast pigment content and net photosynthesis, indicative of a dose-dependent phytotoxicity. The concurrent presence of numerous phenolic compounds in the extracts of this species suggests the involvement of secondary metabolites in the disruption of chloroplast function [50]. Analogous effects were observed in experiments with *Populus* spp., where accumulation of phenolic acids derived from the root system and rhizosphere of *Populus* × *euramericana* (Dode) Guinier led to a decline in photosynthetic pigment content and reduced productivity of acceptor plants [54]. This phenomenon is associated with inhibition of enzymes of the tetrapyrrole biosynthetic pathway and accelerated chlorophyll degradation induced by elevated ROS levels, resulting in destabilisation of protein–pigment complexes of thylakoid membranes [28]. As a consequence, the photochemical potential of chloroplasts is diminished, leading to suppression of the photosynthetic process [28,50].

A key element of the allelopathic phytotoxicity of angiosperm trees is the destabilisation of photosystem II (PSII) function, leading to restricted electron flow and a decline in photochemical efficiency. Treatment of *V. radiata* seedlings with *E. camaldulensis* leaf extracts resulted in reduced chlorophyll fluorescence, indicative of damage to the PSII reaction centre, and enhanced photoinhibition. This phenomenon is accompanied by a concurrent decline in net photosynthetic efficiency, suggesting a close coupling between PSII function and photosynthetic pigment availability [50]. In the case of *Populus* spp., phenolic acids were shown to affect the expression of genes associated with PSI and PSII, leading to restricted ATP and NADPH synthesis and impairment of CO_2_ assimilation. Simultaneously, enhanced oxidative stress and thylakoid membrane damage were observed, indicating a synergistic interplay between photochemical and redox mechanisms in response to allelopathic stress [26]. Particularly potent effects are exerted by juglone—the naphthoquinone allelochemical characteristic of *Juglans* spp.—which directly inhibits net photosynthesis in *C. sativus* seedlings, confirming the high sensitivity of PSII to naphthoquinone compounds [49].

Allelochemicals released by angiosperm trees exert a significant influence on plant gas-exchange parameters, leading to reductions in net photosynthesis (Pn), stomatal conductance (Gs), and transpiration (Tr). A concurrent increase in intercellular CO_2_ concentration (Ci) is observed, indicating that, despite restricted gas diffusion through the stomata, CO_2_ is not being efficiently utilised in assimilatory processes. The restriction of photosynthesis thus does not arise from stomatal limitation per se, but rather from processes occurring within the leaf mesophyll. These include decreased activity of Calvin cycle enzymes, impaired regeneration of CO_2_ acceptors, and disruptions in the functioning of the chloroplast electron-transport chain, collectively resulting in reduced carbon assimilation efficiency, despite CO_2_ availability within the leaf lamina [50,55]. In the case of *E. camaldulensis* extracts, the decline in Pn, Gs, and Tr in *V. radiata* seedlings correlated with a reduction in chlorophyll content, indicating a coupling between disturbances in chloroplast pigment levels and stomatal functioning [50]. Similar phenomena were observed in experiments with *Populus* × *euramericana*, where phenolic acids accumulating in the root tissues of acceptor plants caused marked restriction of CO_2_ assimilation and increased cell membrane damage, pointing to the co-occurrence of photosynthetic disruption at both biochemical and structural levels [55]. In numerous cases, a hormetic response has also been observed, in which low allelochemical concentrations may transiently stimulate gas exchange, whilst higher doses result in its pronounced inhibition [23]. This phenomenon reflects the non-linear character of the physiological response of acceptor plants to allelopathic stress [56].

Exposure of plant cells to allelochemicals results in the induction of oxidative stress, leading to lipid peroxidation, destabilisation of cell membranes, and disruption of chloroplast ultrastructure [27,50]. In leaf tissues of *V. radiata* seedlings treated with *E. camaldulensis* leaf extracts, elevated malondialdehyde (MDA) levels were detected, consistent with intensified lipid peroxidation and loss of cell membrane integrity. Concurrent proline accumulation was observed, interpreted as an adaptive response to oxidative stress [50]. Acceptor plant tissues exposed to phenolic acids derived from *Populus* spp. accumulated these compounds, leading to elevated ROS levels and disturbances in antioxidant enzyme activity, resulting in destabilisation of chloroplast function and a decline in photosynthetic efficiency [28]. Furthermore, it has been demonstrated that extracts from angiosperm trees containing allelochemicals may induce chloroplast deformation, thylakoid reorganisation, and activation of programmed cell-death pathways, representing the terminal stage of cellular damage [27]. Consequently, oxidative stress constitutes a central integrating node for phytotoxic effects at the cellular and subcellular levels [16,27].

In summary, exposure of acceptor plant tissues to allelochemicals emitted by angiosperm trees results in diminished CO_2_ assimilation efficiency, reduced biomass accumulation, and disruption of redox homeostasis, which directly translates into a decline in the competitive fitness of acceptor plants under allelopathic pressure [16,50]. At the ecosystem level, this mechanism may influence the structure of plant communities and succession dynamics, particularly in habitats dominated by angiosperm tree species with high phytotoxic potential [26,54].

### 3.3. Effects of Allelochemicals Emitted by Angiosperm Trees on the Respiration of Acceptor Plants

**Figure 5:**
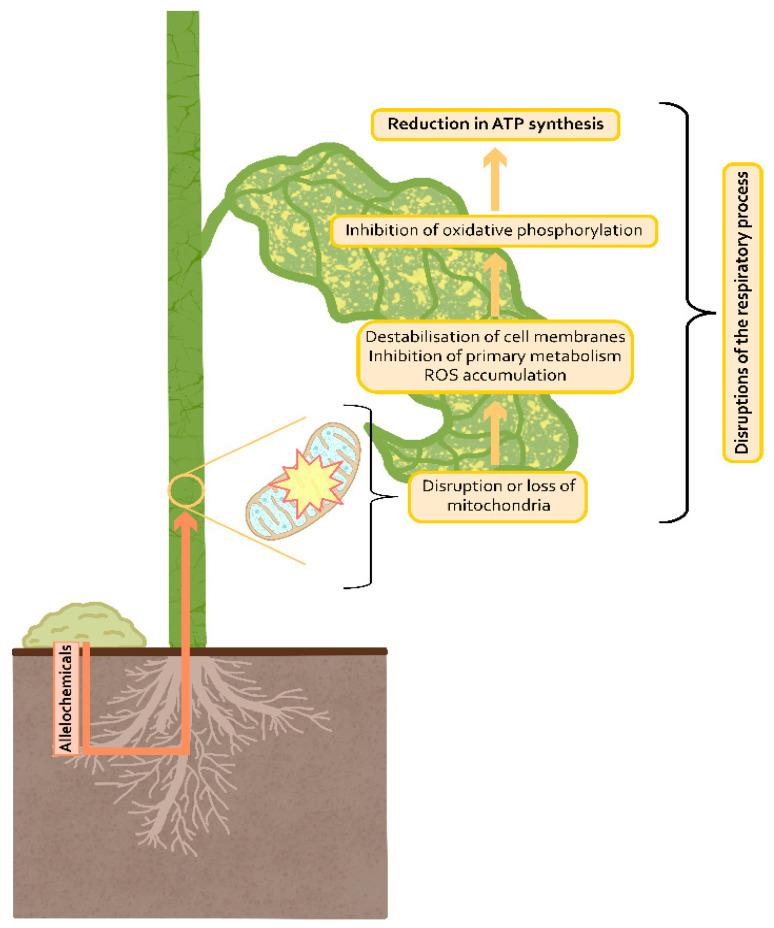
Conceptual summary of the reported effects of allelochemicals on the respiratory processes of acceptor plants, based on evidence synthesised in this systematic review. Allelochemicals released into the rhizosphere are absorbed by the roots and interfere with mitochondrial integrity and function in acceptor plants. Mitochondrial disruption leads to the destabilisation of cellular membranes, inhibition of primary metabolism, and accumulation of reactive oxygen species (ROS). These changes impair oxidative phosphorylation, resulting in reduced ATP synthesis and disruption of cellular respiration. Collectively, these physiological and biochemical alterations compromise energy production, contributing to impaired growth and reduced plant performance. The figure presents a conceptual model integrating the principal mechanisms consistently reported across the studies included in this systematic review.

Allelochemicals emitted by angiosperm trees give rise to multi-level disruptions of the respiratory process in acceptor plants (Figure 5), encompassing concurrent mitochondrial inhibition, destabilisation of cell membranes, induction of oxidative stress, and restriction of primary metabolism. The intensity of these effects has been shown to depend upon allelochemical concentration, tissue type, and chemical class; however, the common denominator remains disruption of cellular energy homeostasis and reduced ATP production efficiency [26,29,57]. Such coupled interactions indicate that the allelopathy of angiosperm trees may function as a systemic modulator of plant energy balance across diverse ecosystems.

One of the key mechanisms underlying the activity of allelochemicals emitted by angiosperm trees is impairment of mitochondrial function and electron transport chain activity, leading to disruption of oxidative phosphorylation. Treatment of plant tissues with p-hydroxybenzoate accumulating in the rhizosphere of *Populus* spp. was shown to alter the expression of genes encoding components of the mitochondrial electron transport chain, resulting in reduced ATP synthesis and elevated ROS production. Disruption of the balance between electron flow and electron reduction led to amplification of oxidative stress, further deepening the energy deficit in acceptor plant cells [26]. This mechanism indicates that inhibition of mitochondrial respiration may constitute the basis of the disintegrating action of numerous phenolic allelochemicals from angiosperm trees. A comparable effect was observed upon exposure of acceptor plant tissues to juglone—the principal allelochemical occurring in the leaves, roots, and other tissues of *Juglans* spp. This compound caused a significant reduction in oxygen uptake and inhibition of respiration in root and leaf tissues of acceptor plants, with the effect intensifying with increasing concentration. Furthermore, juglone inhibited the activity of membrane-bound H^+^-ATPase, leading to disruption of the proton gradient essential for the coupling of transport and energy metabolism. As a consequence, reduced growth, decreased dry mass accumulation, and diminished water uptake were observed, indicating the systemic nature of the energy dysfunction induced by angiosperm tree allelochemicals [58].

In parallel, an important role in respiratory disruption is played by the destabilisation of cell and mitochondrial membranes, which amplifies metabolic dysfunction. Treatment of acceptor plant tissues with *Juglans regia* L. leaf extract was demonstrated to inhibit the respiratory process and disrupt gas exchange—including alterations in CO_2_ concentration—indicative of deregulation of transport processes dependent upon membrane integrity [59]. In other experimental systems, phenols and flavonoids were shown to act directly upon mitochondrial membranes, causing loss of their structural integrity and, consequently, increased permeability [16]. Application of concentrated allelochemicals resulted in an 80–90% decline in antioxidant enzyme activity and intense ion leakage, unequivocally indicating advanced disintegration of cell membranes [29]. These secondary disturbances also compromise respiratory efficiency through disruption of electrochemical gradients within the cell.

A critical element of the plant response to allelochemical exposure is the induction of oxidative stress, which acts both as a primary toxic effect and as a mechanism amplifying mitochondrial damage. Elevated lipid peroxidation—as measured by MDA content—concurrent with a decline in the activity of antioxidant enzymes including superoxide dismutase (SOD) and catalase, has been observed across numerous experimental systems [29,60]. Treatment of *Bromus japonicus* Thunb. with extracts from angiosperm trees containing allelochemicals resulted in over 80% reduction in catalase activity and a marked decrease in protein content, indicative of profound disruption of redox homeostasis and cellular metabolism [29]. It was further demonstrated that decomposition products of *Cinnamomum camphora* (L.) J. Presl leaf litter may reduce the antioxidant capacity of plants growing in the vicinity of the allelochemical donor and affect photochemical parameters, pointing to a close coupling between energy metabolism and oxidative stress [60]. Excess ROS leads both to peroxidation of membrane lipids and to inactivation of mitochondrial proteins, thereby secondarily deepening respiratory inhibition.

An important, frequently overlooked mechanism is the restriction of metabolic substrate availability for respiration, arising from inhibition of primary metabolism. Exposure to angiosperm tree extracts containing phenols, alkaloids, and acetogenins has been shown to reduce the contents of proteins, amino acids, and carbohydrates in plant tissues. The concurrent inhibition of enzymes critical to primary metabolism and disruption of nucleic acid stability were observed, indicating global suppression of biosynthesis at the whole-organism level [57]. As a consequence, the diminished pool of metabolic substrates further amplifies the ATP deficit arising from mitochondrial inhibition, generating a synergistic effect.

At the ultrastructural level, the allelopathy of angiosperm trees leads to degeneration of cellular organelles, particularly mitochondria. Loss of organelles in root-tip cells and the presence of deformed mitochondria and plastids have been reported [61]. In other experimental systems, a reduction in mitochondrial number and structural degradation were observed, correlating with decreased respiratory intensity and destabilisation of cellular metabolism [16]. These changes are partially irreversible at higher allelochemical concentrations, and result in permanent growth inhibition and impairment of physiological function in acceptor plants.

In summary, inhibition of respiration in acceptor plants under the influence of allelochemicals derived from angiosperm trees arises from the synergistic action of several mechanisms: (i) direct inhibition of the mitochondrial electron transport chain, (ii) destabilisation of cell and mitochondrial membranes, (iii) induction of oxidative stress, and (iv) restriction of metabolic substrate availability. The consequences of these processes include a systemic energy deficit, leading to disruption of redox homeostasis, reduced ATP synthesis, and suppression of plant growth [26,29,57].

### 3.4. Water Relations of Acceptor Plants Under Exposure to Allelochemicals from Angiosperm Trees

**Figure 6:**
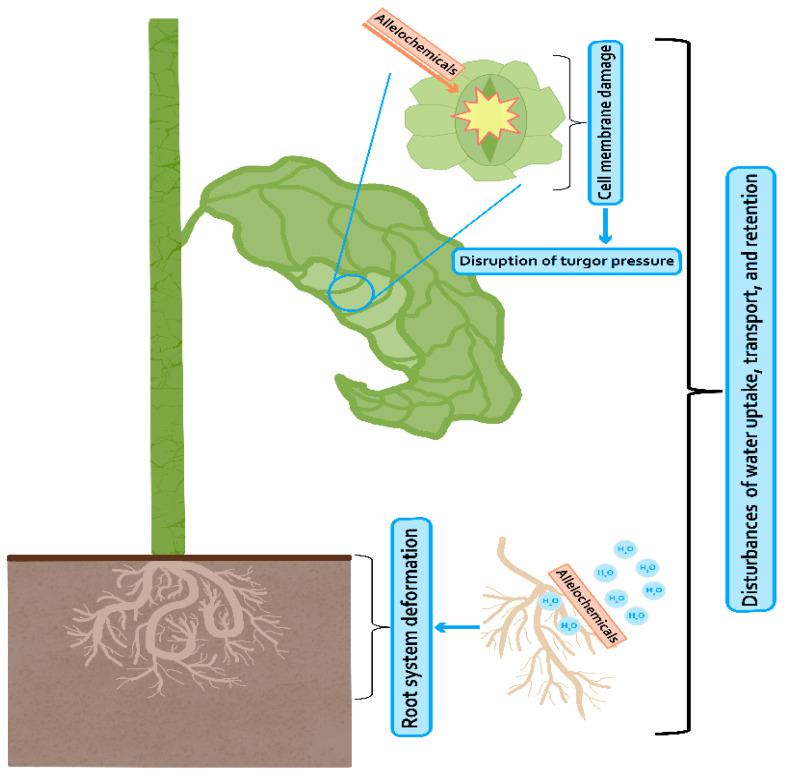
Conceptual summary of the reported effects of allelochemicals on water relations in acceptor plants, based on evidence synthesised in this systematic review. Allelochemicals released into the rhizosphere and aerial environment can affect plant water relations at multiple levels. In leaves, they induce cellular membrane damage and lead to a reduction in cell turgor pressure, which ultimately impairs leaf structure and function. In roots, allelochemicals contribute to root system deformation, thereby limiting the capacity for water uptake. Collectively, these alterations disrupt water absorption, transport, and retention within the plant, resulting in impaired physiological homeostasis and reduced plant performance. The figure presents a conceptual model integrating the principal mechanisms consistently reported across studies included in this systematic review.

The allelopathic action of compounds released by angiosperm trees leads to multi-level disruptions of water relations in acceptor plants, encompassing stomatal functioning, cell membrane integrity, tissue osmotic potential, and root system architecture (Figure 6). A key element of this action is the coupling between water relation disturbances and oxidative stress, resulting in restricted transpiration and disruption of whole-plant water balance [62,63,64]. Disruption of acceptor-plant water relations is a multi-stage process dependent upon allelochemical concentration, encompassing both rapid responses manifested in stomatal movements and long-term structural changes in root tissues [65,66]. 

One of the primary mechanisms underlying water relation disturbances is the restriction of stomatal conductance and transpiration, arising from direct or indirect modulation of stomatal functioning. Treatment of acceptor plants with root extracts of *Casuarina equisetifolia* L. resulted in a significant reduction in stomatal conductance and transpiration, indicating the induction of water loss-limiting mechanisms in response to allelopathic stress [63]. Analogous effects were observed in plants exposed to leaf litter of *Cinnamomum septentrionale*, where alterations in gas exchange parameters indicated disturbances in stomatal functioning and secondary restriction of water transport within the plant [52]. Application of *J. regia* leaf extracts resulted in a reduction in stomatal conductance, leading to restricted transpiration and disrupted gas exchange, consistent with a systemic character of the response [59].

In parallel, an important mechanism is damage to cell membrane integrity, leading to uncontrolled electrolyte leakage and disruption of water retention in plant tissues. Angiosperm tree allelochemicals have been demonstrated to increase cell membrane permeability, resulting in loss of turgor and reduced capacity of cells to maintain water homeostasis [67]. Changes in membrane integrity, assessed on the basis of electrolyte conductivity, confirm increased structural cell damage and secondary disruption of water relations [68]. Exposure of acceptor plants to phenolic allelochemicals resulted in increased cellular damage, which further amplified water loss and destabilised the whole-plant water balance [69].

An important element of water relation regulation is also the cellular osmotic potential, which undergoes significant disturbance under allelopathic stress conditions. Treatment of white mustard (*Sinapis alba* L.) seeds with a 1% aqueous extract prepared from *Acer pseudoplatanus* L. leaves resulted in the strongest inhibition of germination among all the maple species tested, reducing relative germination by 55% compared with the control—a stronger effect than that of the reference extract from *Juglans nigra* L. The inhibition of germination was significantly correlated with the osmotic potential of the applied extracts, with the *A. pseudoplatanus* extract showing one of the lowest osmotic potentials among the tested maple species, which directly constrained the water potential gradient required for seed imbibition, and points to a substantial involvement of osmotic stress in the observed phytotoxic effect. Significant correlations were also found for other physico-chemical parameters of the extracts, including flavonoid, total phenolic, and tannin contents, indicating that osmotic stress acted in conjunction with additional allelopathic mechanisms. [70]. In other experimental systems, exposure of acceptor plant tissues to angiosperm tree allelochemicals was accompanied by changes in cellular osmotic potential and accumulation of osmoprotectants—including proline and soluble sugars—representing a compensatory mechanism enabling partial stabilisation of water relations [38,71]. Simultaneously, application of an aqueous extract from fig tree (*Ficus carica* L.) stems, consisting mainly of phenolic compounds (68.2% of the extract) and terpenoids, at low concentrations (5.0–10.0 g/L) led to a transient improvement in water-binding capacity and growth in the recipient plant *Siraitia grosvenorii*, which the authors attribute to an increase in cellular osmotic pressure and stimulation of respiratory enzyme activity, whereas higher concentrations (15.0–25.0 g/L) caused a marked disturbance of growth and physiological parameters in the plant [26,72].

In response to allelopathic stress, plants activate adaptive mechanisms encompassing osmoprotectant accumulation and modulation of redox metabolism. Accumulation of proline and soluble sugars has been shown to support partial stabilisation of osmotic potential and protection of cellular structures against water loss [38,72]. Concurrent modulation of antioxidant enzyme activity—including SOD, peroxidase (POD), ascorbate peroxidase (APX), and catalase (CAT)—has been documented as part of the broader physiological stress response to allelochemical exposure, as shown for *C. equisetifolia* leaf-litter-leachate and *C. septentrionale* leaf-litter-decomposition products [60,63]. While such enzymatic shifts are often interpreted as components of an integrated stress response that may also extend to water relations, direct mechanistic coupling between antioxidant modulation and osmotic adjustment has not been demonstrated within these specific systems. Under conditions of high stress intensity, however, compensatory capacity becomes exhausted, resulting in further deepening of water relation disturbances and collapse of overall metabolism [72].

Root system deformation also plays a significant role in water relation disruption, resulting in reduced efficiency of water uptake from the substrate. In numerous acceptor species—including *Lactuca sativa* L., *Solanum lycopersicum* L., and *Festuca rubra* L.—treated with extract from *Acer ginnala* Maxim. fruit husk layers, root shortening and alterations in root architecture were observed, leading to reduced water absorption capacity and deterioration of plant water balance [73]. Similarly pronounced inhibition of root growth in plants treated with extracts from the bark, seeds, and leaves of *Tamarindus indica* L. indicates secondary restriction of water uptake as a key element of physiological stress [74]. In experiments in which VOCs and wood residues of *Ailanthus altissima* were applied to seeds of acceptor plants, pronounced germination inhibition was observed and linked to the activity of compounds modifying the soil water microenvironment, thereby further restricting water availability [75].

At the whole-plant level, changes in physiological parameters indicative of water stress induction are also observed, including reduced relative water content and tissue osmotic potential. Under exposure to allelopathic extracts, a decline in seedling water content was recorded, indicating disruption of cellular and tissue-water homeostasis. These changes were strongly concentration-dependent, confirming the involvement of osmotic regulation and water retention mechanisms in the plant response [76]. Furthermore, reduction in relative water content correlated with a decline in photosynthetic parameters, indicating a close coupling between water relations and photosynthetic apparatus functioning [64].

An additional factor modulating water relations is the influence of allelochemicals on soil properties and water availability in the soil environment. The presence in the soil of allelochemicals emitted by *Rhus typhina* L. alters soil physicochemical parameters—including organic matter content—thereby affecting water retention and water availability to plants [17]. Under water-deficit stress conditions, allelopathic effects were intensified, indicating a synergistic interaction between water resource limitation and allelochemical presence [77]. In soil systems containing *A. altissima* wood residues, prolonged germination inhibition was observed, potentially associated with persistent alterations in the water properties of the soil environment [75].

In summary, allelopathic disruption of water relations in acceptor plants results from the integrated action of multiple mechanisms encompassing (i) dysregulation of stomatal functioning and restriction of transpiration, (ii) cell membrane damage leading to water loss and loss of turgor, (iii) disruption of osmotic potential and impairment of water homeostasis, and (iv) root system deformation and alterations in soil properties. Allelochemicals derived from angiosperm trees initiate a cascade of physiological disturbances leading to restriction of water uptake, transport, and retention, and, consequently, to growth inhibition and death of acceptor plants [38,60,67,70].

### 3.5. Effects of Allelochemicals Emitted by Angiosperm Trees on Mineral Nutrition of Acceptor Plants

**Figure 7:**
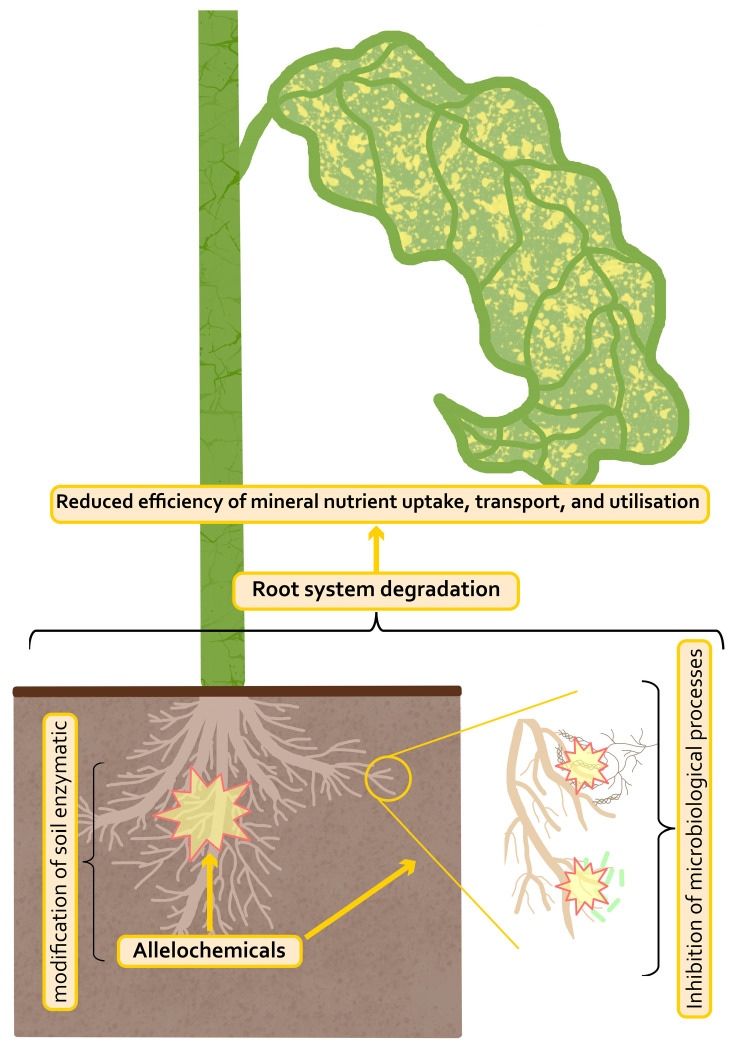
Conceptual summary of the reported effects of allelochemicals on mineral nutrition in acceptor plants, based on evidence synthesised in this systematic review. Allelochemicals released into the rhizosphere negatively affect soil–plant interactions by altering soil enzymatic activity and inhibiting key microbial processes. These changes contribute to root system degradation and reduced root functionality. As a consequence, the efficiency of mineral nutrient uptake, transport, and utilisation is decreased. Collectively, these disturbances in below- and above-ground processes impair plant nutritional status, leading to reduced growth and overall plant performance. The figure presents a conceptual model integrating the principal mechanisms consistently reported across studies included in this systematic review.

Allelochemicals emitted by angiosperm trees govern the functioning of acceptor plants through coupled interactions encompassing modification of the soil environment, disruption of microbial activity, and direct phytotoxic effects on plant physiology (Figure 7). From a systemic perspective, allelopathy constitutes a multi-level regulatory mechanism in which a key role is played both by the chemical activity of secondary metabolites and by their transformation in the rhizosphere and interactions with the soil microbiome, ultimately translating into restricted growth and impaired mineral homeostasis in acceptor plants [16].

The action of allelochemicals emitted by angiosperm trees on plant mineral nutrition is largely based on impairment of the acceptor plant root system, which directly reduces its capacity for mineral nutrient uptake. Compounds such as mimosine and phenols—released by *Leucaena leucocephala* from leaves and roots—induce damage to root apical meristems, meristematic necrosis, and inhibition of root hair formation, leading to a significant reduction in root absorptive-surface area. The consequences of these changes include mechanical restriction of root–soil contact and a decline in the efficiency of macro- and micronutrient uptake [78,79].

In parallel, allelochemicals emitted by angiosperm trees profoundly disrupt nitrogen cycling in the soil through inhibition of microbiological processes responsible for nitrification. Catechins present in exudates of *L. leucocephala*—from leaves and root litter—inhibit the activity of *Nitrosomonas europaea* Winogradsky, leading to a reduced rate of ammonia oxidation and disruption of the NH_4_^+^/NO_3_^−^ ionic balance [79]. Additionally, phenolic constituents of the litter of woody species—including decomposing leaf litter of *Casuarina equisetifolia* and other arboreal species—reduce ammonium and nitrate ion concentrations and reorganise the functional structure of ammonia-oxidising bacterial communities, indicating a microbiologically mediated restriction of plant-available mineral nitrogen in the soil [80]. An important component of the allelopathic mechanism is also the disruption of biological nitrogen fixation, which further deepens the nitrogen deficit in the plant-soil system. Extracts from *Celtis occidentalis* L. leaves inhibit root nodule formation in acceptor plant roots, restricting the activity of symbiotic diazotrophs, and thereby reducing the input of biologically fixed nitrogen. This mechanism indicates that the action of allelochemicals simultaneously encompasses effects on the soil environment, leading to a multidimensional reduction in nitrogen availability [81].

At the physiological level, direct disruptions of nitrogen assimilation resulting from allelochemical action are observed in acceptor plants. Mimosine present in *L. leucocephala* leaf tissues inhibits nitrate reductase activity in the leaf tissues of acceptor plants, restricting the reduction of nitrates to assimilable forms and their incorporation into amino acid metabolism. Simultaneously, a decline in the activity of enzymes associated with cellular metabolism—including peroxidase and phenylalanine ammonia-lyase (PAL)—is observed, leading to further disruption of metabolic homeostasis and restricted plant growth [82].

A further level of mineral nutrition regulation is the modification of the soil microbiome responsible for organic matter mineralisation. Root extracts of *Rhus typhina* reduce soil organic carbon and total nitrogen contents, whilst simultaneously reducing the metabolic activity of soil microorganisms and their capacity for carbon substrate utilisation [17]. Similarly, compounds released from the decomposing litter of *Cinnamomum migao* H.W.Li—from leaves, branches, seeds, and fruit walls—affect soil enzyme activity and fungal community structure, destabilising the biogeochemical cycling of C and N in the rhizosphere and reducing the efficiency of mineral nutrient cycling [83].

An additional mechanism of action is the effect of allelochemicals on micronutrient bioavailability through metal ion complexation. Mimosine—released in root and leaf exudates of *L. leucocephala*—exhibits the capacity to form stable complexes with Fe(III), modifying its mobility in the soil and competitive availability in the rhizospheric environment. Such chemical interactions lead to disruptions of micronutrient balance that may secondarily affect enzymatic activity and redox processes in acceptor plants [84].

In the soil environment, an important role is also played by the accumulation and transformation of allelochemicals derived from the litter and root exudates of angiosperm trees. In the rhizosphere of *J. regia*—from roots and leaf litter—phenolic compounds, fatty acids, and 5-hydroxy-1,4-naphthoquinone are detected, influencing the sorption, transport, and bioavailability of mineral nutrients [85]. Simultaneously, juglone induces changes in cell wall lignification in acceptor-plant root tissues through activation of phenylpropanoid pathways, restricting ion and water transport and deepening mineral disturbances [86].

The complexity of allelopathic interactions between angiosperm trees and their environment also arises from their effects on soil physicochemical properties and litter decomposition dynamics. Extracts and litter of trees containing compounds with allelopathic potential may alter soil pH and electrical conductivity, directly affecting ion bioavailability and growth conditions for acceptor plants. Concurrent accumulation of phenols and flavonoids in the soil indicates long-term biological activity of these compounds, favouring the maintenance of inhibitory effects over extended time periods [87].

Furthermore, soil enzymatic properties—including the activity of urease, phosphatases, and polyphenol oxidase—are modulated by allelochemicals of arboreal origin, affecting the rate of nutrient mineralisation [88,89]. These disruptions translate into restricted regeneration of available nitrogen and phosphorus pools, secondarily deepening mineral deficits in acceptor plants [88].

In summary, the available evidence indicates that allelochemicals emitted by angiosperm trees affect the mineral nutrition of acceptor plants through an integrated set of mechanisms encompassing root system degradation, inhibition of microbiological processes—including nitrification and mineralisation in the soil—disruption of symbiotic nitrogen fixation, modification of soil enzymatic activity, and direct inhibition of enzymes critical to plant metabolism [17,78,79,81,82,88]. The cumulative effect of these processes is a systemic reduction in the efficiency of mineral nutrient uptake, transport, and utilisation, leading to disrupted growth and impaired functioning of acceptor plants in ecosystems subject to allelopathic pressure from angiosperm trees.

### 3.6. Effects of Allelochemicals Emitted by Angiosperm Trees on the Growth and Development of Acceptor Plants

**Figure 8:**
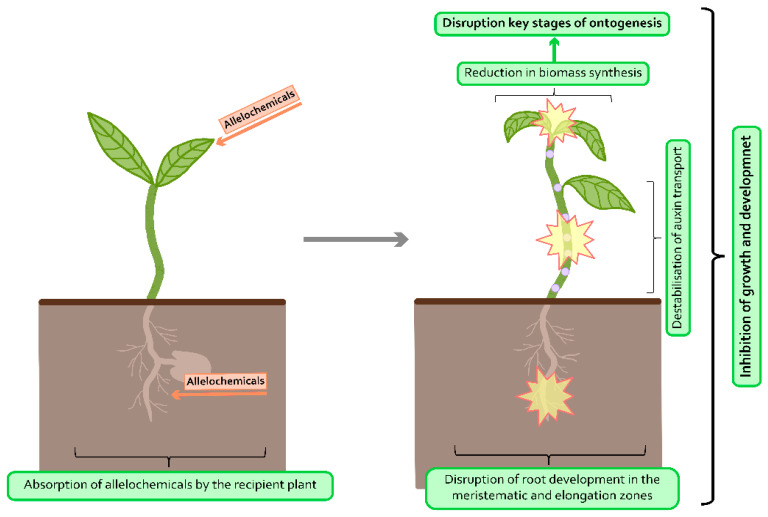
Conceptual summary of the reported effects of allelochemicals on plant growth and development of acceptor plants, based on evidence synthesised in this systematic review. Allelochemicals are absorbed by recipient plants through both roots and shoots, initiating a cascade of physiological and developmental disturbances. These compounds disrupt root growth and development, particularly in meristematic and elongation zones, and interfere with auxin transport and signalling pathways. At the shoot level, allelochemicals impair key stages of ontogenesis and reduce biomass synthesis. Collectively, these effects lead to a general inhibition of plant growth and development. The figure presents a conceptual model integrating the principal mechanisms consistently reported across studies included in this systematic review.

The allelopathic action of compounds released by angiosperm trees constitutes an important factor modifying the growth and development of acceptor plants (Figure 8), affecting key stages of ontogenesis through disruption of cell division, tissue elongation, and assimilate allocation [52,90]. In numerous experimental systems, this effect has been shown to be dose-dependent, with increasing concentrations of litter or leaf extracts leading to progressive reductions in biomass and morphological parameters, indicative of the cumulative nature of chemical stress [91,92]. Particularly pronounced inhibition is observed with respect to root systems—specifically, the apical meristematic and elongation zones—suggesting high sensitivity of root apical meristems to phenols, flavonoids, and other allelochemicals emitted by angiosperm trees [93,94].

In numerous experimental systems in which extracts from allelopathically active angiosperm trees were applied to acceptor plant tissues, inhibition of biomass accumulation and elongation of roots and shoots was observed; the root meristematic and elongation zones represent the most sensitive components of acceptor plants [94]. The concentration-dependence of the allelochemical effect indicates the cumulative nature of cellular damage, encompassing disruption of the cell cycle, energy metabolism, and hormonal homeostasis [94,95]. As a consequence, the regenerative capacity of plants is diminished, and their competitive fitness under allelopathic pressure is reduced [94].

Under exposure to *Cinnamomum septentrionale* leaf litter, significant restriction of maize (*Zea mays* L.) growth was recorded—encompassing reductions in biomass and morphological traits—with the effect intensifying with increasing litter dose [90]. In pot experiments at doses ≥ 40 g per pot, pronounced inhibition of vegetative growth was observed, indicating that the metabolic-tolerance threshold of acceptor plants had been exceeded [91]. Analogous effects were observed in *Z. mays*, *Cucumis sativus*, *and Vigna unguiculata*, where both leaf area and fresh biomass were reduced, reflecting disruption of leaf growth and consequent biomass reduction. These changes point to the systemic nature of allelochemical action [52].

Upon exposure of acceptor plants to *Cinnamomum camphora* litter, significant inhibition of seedling growth and disruption of generative development were observed—including delayed flowering and reduced flower number—indicative of multi-level disturbances in acceptor plant growth and development. This effect is associated with the presence of volatile monoterpenes and sesquiterpenes, which may interfere with the hormonal regulation of acceptor plants, particularly the auxin–cytokinin signalling pathways responsible for the coordination of growth and tissue differentiation. Concurrent restriction of root system development—encompassing both primary and lateral roots—and reduction in biomass were observed, indicating disruption of assimilate partitioning between organs [42].

Decomposition of *C. camphora* litter under soil conditions leads to significant growth restriction in numerous plant species, including lettuce (*Lactuca sativa* L.), aubergine (*Solanum melongena* L.), and cabbage (*Brassica oleracea* L.), encompassing reductions in leaf number and area and inhibition of root elongation. In the early stages of exposure, elevated malondialdehyde (MDA) levels and accumulation of soluble sugars in leaf and root tissues are observed, indicative of oxidative-stress induction and secondary disorganisation of cellular energy metabolism. Simultaneously, a decline in soluble protein content in leaf and root tissues suggests disruption of protein biosynthesis and destabilisation of anabolic processes, directly translating into reduced growth rate. Additionally, changes in the soil microbiome —including disruption of rhizospheric C and N biomass—may indirectly restrict mineral nutrient availability and intensify the phytotoxic effect [60].

Extracts and litter of *Casuarina equisetifolia* exhibit potent inhibitory effects on germination and seedling growth of crop species, including maize (*Z. mays*), lentil (*Lens culinaris* Medik.), and wheat (*Triticum aestivum* L.), indicating a broad phytotoxic spectrum of this class of allelochemicals. The observed shortening of roots and shoots results from disruption of cell division and restricted cell elongation in the meristematic zones of roots and shoots, representing a typical response to the action of phenols and other secondary metabolites [96]. Furthermore, the presence of specific acids and root metabolites in root exudates may induce autotoxicity, leading to restricted regeneration and restricted population renewal in the soil environment [97].

In experiments with *Impatiens balsamina* L., *Cinnamomum japonicum* Siebold ex Nees litter caused inhibition of vegetative growth and disruption of flowering, indicating interference with the regulation of generative development [50]. Similar effects were observed in experiments with invasive plant species, where aqueous extracts of angiosperm trees strongly restricted the growth of acceptor species, with the intensity of inhibition increasing with allelochemical concentration [68,94]. The observed effects were attributable to disruption of root- and shoot-meristem functioning, destabilisation of auxin transport, and restriction of biomass synthesis arising from energy deficits [36,93]. Consequently, the allelopathy of angiosperm trees constitutes a factor that regulates acceptor plant growth and development across multiple levels, integrating interactions at the cellular, tissue, and whole-organism levels [23,50].

## 4. Discussion

The findings of the present review indicate that the allelopathic action of compounds emitted by angiosperm trees on acceptor plants is multi-level and systemic in nature, encompassing concurrent disruption of photosynthesis, respiration, water relations, mineral nutrition, and growth and development. This pattern of physiological response is not coincidental—it arises from the structural and functional properties of allelochemicals which, by virtue of their lipophilicity, redox reactivity, and capacity to chelate metal ions, act upon multiple cellular processes simultaneously [33,41]. The key conclusion emerging from the analysis of the available literature is therefore that the allelopathy of angiosperm trees should not be regarded as a sum of independent phytotoxic effects, but rather as an integrated systemic response in which disruption of one physiological process inevitably entails destabilisation of the others [16,29].

Comparison of physiological responses among different groups of acceptor plants did not reveal consistent evidence indicating that a particular functional group, such as crops, weeds, herbaceous species, or woody plants, exhibits universally greater sensitivity to allelochemicals released by angiosperm trees. The majority of available studies have focused on herbaceous and crop species, including *Zea mays*, *Cucumis sativus*, *Vigna* spp., *Triticum aestivum*, *Lactuca sativa*, and other economically important species, frequently during germination or early seedling development, which limits direct comparisons among broader plant functional groups [50,52,60,63,73,90,96].

Despite the lack of clear differences between taxonomic or functional groups, the reviewed evidence suggests that susceptibility to allelochemical stress is strongly associated with physiological and ecological traits of acceptor plants, rather than with their classification as crops, weeds, or other plant categories. Species characterised by rapid growth, high metabolic activity, intensive root development, and high demand for efficient water and nutrient acquisition may be particularly vulnerable because allelochemicals commonly affect root meristem activity, cell elongation, photosynthetic efficiency, respiration, and mineral uptake, simultaneously [16,26,27,28,78,93,94].

Across the physiological processes analysed in this review, the most consistent pattern was the high sensitivity of young tissues, especially root systems, which represent primary targets of phenolic compounds, flavonoids, naphthoquinones, and other tree-derived allelochemicals. Inhibition of root elongation, reduction of root absorptive capacity, and disruption of root–soil interactions were repeatedly reported in different acceptor species, including both crop plants and non-crop herbaceous species [73,78,93,94,96]. Similarly, differences in antioxidant capacity, osmotic regulation, and interactions with the rhizospheric microbiome may contribute to species-specific variation in tolerance to allelochemical exposure [17,38,63,71,83].

Therefore, the available evidence suggests that the response of acceptor plants to angiosperm tree allelochemicals is determined primarily by functional physiological traits and adaptive capacity, rather than by taxonomic affiliation alone. However, the current literature remains insufficient for predicting sensitivity patterns among broad plant groups, particularly due to the limited number of comparative studies, including multiple acceptor species representing different ecological strategies under standardised experimental conditions.

A central role in the integration of allelopathic effects is played by oxidative stress. Analysis of the data presented across the individual sections of this review indicates that induction of oxidative stress through reactive oxygen species (ROS) accumulation constitutes the common denominator of disturbances affecting photosynthesis, respiration, water relations, and growth in acceptor plants. Redox-active allelochemicals—principally juglone and other naphthoquinones, as well as phenols and flavonoids—generate ROS in both chloroplasts and mitochondria, leading to peroxidation of membrane lipids, inactivation of antioxidant enzymes, and destabilisation of organelle ultrastructure [24,26,27]. Elevated malondialdehyde (MDA) levels—as a marker of lipid peroxidation—alongside concurrent declines in catalase and superoxide dismutase (SOD) activity have been demonstrated in the leaf tissues of allelochemical-exposed seedlings [29,50]. These findings are consistent with the hypothesis that oxidative disintegration of biological membranes constitutes the primary mechanism of phytotoxicity of angiosperm tree allelochemicals, and that all other observed effects—disruption of electron transport, stomatal closure, inhibition of mineral ion uptake, and suppression of cell division—represent its secondary consequences [16,27,28].

It should be emphasised that oxidative stress induced by angiosperm tree allelochemicals should not be considered exclusively as either an initiating mechanism or a secondary consequence of physiological disruption. Current evidence indicates that ROS accumulation represents a dynamic and bidirectional component of the allelopathic response. Certain redox-active compounds, particularly naphthoquinones such as juglone and selected phenolic metabolites, may directly promote ROS generation through interference with mitochondrial respiration, chloroplast electron transport, and cellular redox balance [24,25,26,27]. At the same time, oxidative stress may arise as a consequence of earlier physiological disturbances, including impaired photosynthesis, mitochondrial dysfunction, membrane destabilisation, and altered water and mineral relations [26,28,29,64]. Therefore, ROS function as amplifying mechanisms within the allelopathic stress network: initial oxidative imbalance promotes lipid peroxidation, organelle damage, and metabolic disruption, which subsequently intensify oxidative pressure and further impair plant functioning [16,27,29]. This feedback relationship may explain why chemically diverse groups of allelochemicals ultimately converge on similar physiological outcomes in acceptor plants [33,41].

A further interpretative challenge in allelopathy research is the distinction between specific allelochemical effects and general phytotoxic-stress responses. Physiological alterations frequently reported after exposure to angiosperm tree-derived compounds, including reduced photosynthetic efficiency, disruption of water relations, membrane damage, and ROS accumulation, are not exclusive signatures of allelopathy, and may also occur during other forms of environmental stress. Therefore, physiological responses were considered indicative of allelochemical action primarily when supported by evidence of exposure to identified compounds, chemically characterised extracts, litter-derived metabolites, or root exudates, together with appropriate controls and concentration-dependent responses [26,28,29,50,64]. Nevertheless, the convergence of allelopathic effects with general-stress pathways represents an inherent limitation of current allelopathy research and highlights the need for integrative approaches combining chemical analysis with physiological, biochemical, and molecular assessments.

Equally important is the concentration-dependent nature of allelopathic interactions, which in many plant–plant systems does not follow a linear pattern. This phenomenon, termed hormesis, is characterised by stimulatory effects of low allelochemical concentrations and inhibitory effects of higher concentrations, and has been widely documented in the allelopathic literature since the classical work of Molisch and Rice [2,7]. Within the present review, this pattern was observed in several systems involving angiosperm trees as donors. It was most clearly demonstrated in a study of the effect of *Ficus carica* stem extract on *Siraitia grosvenorii*, where low extract concentrations (5.0–10.0 g/L) increased seed germination, biomass, and photosynthetic parameters, whereas higher concentrations (15.0–25.0 g/L) caused their marked inhibition [98]. A similar pattern, though confined to the level of the antioxidant response and not extending to germination or growth (which were inhibited monotonically with increasing concentration), was demonstrated for the aqueous leaf extract of *Quercus mongolica* acting on *Brassica pekinensis* and *Raphanus sativus*: catalase, superoxide dismutase, and peroxidase activities increased at low extract concentrations (0.02–0.04 g/mL), before being inhibited at the highest concentrations. A partially convergent pattern of germination stimulation at low concentration was also observed in response to *Casuarina equisetifolia* leaf litter leachate [63]. Given the limited number of studies within the present review that systematically tested a broad range of allelochemical concentrations from angiosperm tree donors, assessment of the true prevalence of this phenomenon, and of the physiological level at which it most commonly manifests, requires further research encompassing a wider range of donor and recipient species. This phenomenon has significant implications from both ecological and methodological perspectives. From an ecological standpoint, it suggests that the allelopathic action of angiosperm trees under natural conditions may be more subtle and complex than would be inferred from bioassay results obtained at high extract concentrations. From a methodological perspective, it highlights the necessity of employing a broad concentration range in allelopathic experiments and of incorporating ecologically realistic soil-allelochemical concentrations when designing experimental systems. Failure to account for the hormetic effect in the interpretation of results may lead to overestimation or underestimation of the actual allelopathic potential of the tree species under investigation [91,99,100].

A significant problem emerging from the analysis of the available literature is the dominance of the phenomenological over the mechanistic approach. A substantial proportion of the cited studies documents allelopathic effects at the level of biomass, root length, or germination parameters [52,90,96], without attempting to identify the molecular targets of allelochemical action or the signalling pathways activated in response to allelopathic stress. Whilst this approach is valuable from the perspective of documenting the phenomenon, it limits the possibility of formulating general principles, and hinders the comparison of results across experimental systems. In light of the available data, however, several physiological processes may be identified as particularly sensitive to the action of angiosperm tree allelochemicals: photosystem II (PSII) functioning [49,50], membrane-bound H^+^-ATPase activity [58], nitrate reductase activity [84], and the integrity of mitochondrial and chloroplast membranes [16,29]. These processes should constitute priority targets for future mechanistic investigations.

A promising avenue for overcoming the phenomenological limitations outlined above lies in the application of multi-omics approaches, which remain markedly under-represented in the allelopathic literature reviewed here. Of the studies discussed throughout this review, only a small minority extend beyond classical physiological and biochemical endpoints to interrogate the molecular basis of allelochemical action directly. A notable exception is the integration of small-RNA, degradome, and transcriptome sequencing performed in *Populus* × *euramericana* exposed to para-hydroxybenzoic acid, which identified specific microRNA-target gene regulatory modules underlying the suppression of photosynthetic gene expression [26]. Comparable transcriptomic approaches have also been applied to characterise the genome-wide response of recipient plants to juglone and related naphthoquinones [8], revealing coordinated downregulation of genes associated with photosystem assembly and upregulation of stress-responsive transcription factors. Such studies illustrate the explanatory power of omics-based methods in moving beyond the documentation of phytotoxic symptoms, towards the identification of the regulatory networks that generate them.

The near-absence of metabolomic and proteomic studies within the angiosperm tree-allelopathy literature constitutes a particularly significant gap. Metabolomic profiling of acceptor plant tissues following allelochemical exposure would allow the dynamic mapping of perturbations to primary and secondary metabolism—including shifts in the phenylpropanoid, tricarboxylic acid, and antioxidant pathways—which are currently inferred only indirectly from individual-enzyme activity assays. Similarly, proteomic approaches could resolve the discrepancy, noted repeatedly throughout this review, between transcriptional and enzymatic responses to allelochemical stress, by directly quantifying changes in the abundance and post-translational modification of key stress-response proteins. The application of epigenomic methods, including DNA methylation- and chromatin-accessibility profiling, represents a further frontier that has not yet been explored in the context of angiosperm tree allelopathy, despite growing evidence from the broader plant-stress literature that epigenetic reprogramming contributes to both the immediate phytotoxic response and the priming of acceptor plants for subsequent allelochemical exposure.

Beyond the acceptor plant itself, multi-omics approaches also hold considerable promise for elucidating the role of the rhizospheric microbiome as a mediator of allelopathic interactions, a dimension addressed only superficially in the majority of studies reviewed here. Metagenomic and metatranscriptomic characterisation of soil microbial communities exposed to angiosperm tree allelochemicals would enable direct assessment of the functional shifts in nitrogen- and carbon-cycling pathways inferred indirectly from enzymatic and chemical soil analyses in this review [17,83]. Integration of plant-level transcriptomic or metabolomic data with parallel microbiome sequencing—a multi-omics, cross-kingdom approach increasingly applied in plant–microbe interaction research more broadly—would represent a substantial advance over the largely univariate, single-organism perspective that currently characterises this field. The adoption of such integrative frameworks is likely to be essential for resolving the systemic, multi-level nature of angiosperm tree allelopathy proposed in this review, and for distinguishing direct allelochemical effects on acceptor plant physiology from those mediated indirectly via modification of the soil microbial community.

Comparison of the effects exerted by individual allelochemical classes reveals clear differences in their biological activity profiles. Naphthoquinones—primarily juglone—are distinguished by exceptionally high phytotoxic potential arising from their redox properties, which enables ROS generation [24,25]. Phenolic acids and flavonoids exhibit a broader spectrum of action, affecting biological membranes, enzymatic activity, mineral nutrition, and water transport, simultaneously [28,68]. VOCs, in contrast, constitute a distinct category with respect to their mode of action—exerting their effects via the atmospheric pathway without obligatory involvement of the soil environment, thereby being subject to different a ja environmental and seasonal determinants [48,101]. The diversity of activity profiles across allelochemical classes suggests that under natural conditions, allelopathic effects arise from the synergistic or additive action of compound mixtures, rather than from the activity of a single metabolite [33,41].

The synthesis of the available evidence indicates that certain allelochemical classes and angiosperm tree taxa appear repeatedly across studies as particularly important contributors to physiological disruption in acceptor plants. Among these, naphthoquinones, especially juglone produced by species of the genus *Juglans*, represent one of the most consistently characterised groups, due to their strong redox activity and ability to induce oxidative stress, mitochondrial dysfunction, impairment of photosynthetic processes, and growth inhibition [24,25,49,58].

Phenolic compounds and phenolic acids represent another dominant class of tree-derived allelochemicals, being frequently reported in studies involving *Populus*, *Eucalyptus*, *Cinnamomum*, and other angiosperm taxa. Their broad biological activity reflects their capacity to simultaneously affect photosynthetic performance, membrane stability, nutrient acquisition, and plant–soil interactions [26,28,50,54,60,83]. In addition, compounds such as mimosine and catechins produced by *Leucaena leucocephala* appear particularly relevant, due to their effects on root development, nitrogen cycling, and mineral nutrition processes [78,79,80,81,82,83,84].

However, these patterns should not be interpreted as evidence of universal toxicity associated with particular chemical classes or tree taxa. The magnitude and physiological consequences of allelopathic effects remain strongly dependent on allelochemical concentration, environmental transformation processes, exposure pathway, and the physiological characteristics of the acceptor plant [33,41].

A conspicuous gap in the available literature is the insufficient representation of field studies relative to laboratory and pot experiments. The majority of cited works are based on bioassays conducted under controlled conditions, employing aqueous- or organic-plant extracts. In forest ecosystems, however, allelochemicals are subject to complex transformation processes in the soil—including adsorption onto mineral particles and organic matter, microbial degradation, and photodegradation—which may both reduce and enhance their bioavailability and phytotoxicity [24,40]. The rhizospheric microbiome plays a particularly important role as a regulator of allelochemical biological activity—phenolic compounds accumulating in the soil have been shown to modify the structure and activity of soil microorganisms responsible for nitrogen and carbon cycling [17,83], thereby creating a dynamic, mutually regulatory network of interactions. Extrapolation of laboratory results to field conditions, therefore, requires caution, and should be verified through investigations conducted in situ [2].

One of the limitations of the current state of knowledge regarding tree allelopathy is the considerable heterogeneity of experimental approaches used in allelopathy research. Differences in the source of allelochemicals, their application methods, concentration ranges, and plant growth conditions may contribute to variability in the reported effects. Despite this variability, recurring patterns of physiological responses can be identified in the available literature, including alterations in photosynthesis, water relations, energy metabolism, mineral nutrient acquisition, and growth regulation. Therefore, the present review does not focus on comparing the magnitude of allelopathic effects among different experimental systems, but, rather, on integrating current knowledge regarding the physiological mechanisms responsible for the action of tree-derived allelochemicals.

Future studies should aim to combine detailed physiological and biochemical analyses with more standardised experimental approaches, which would improve comparability among studies and facilitate a more precise understanding of the ecological relevance of allelopathic interactions under natural conditions.

The findings of this review also point to considerable variation in the sensitivity of acceptor species to angiosperm tree allelochemicals. Crop plants of the families *Fabaceae*, *Cucurbitaceae*, and *Poaceae* exhibit variable degrees of sensitivity, depending both on the chemical composition of the donor’s allelochemicals and on the detoxification capacity of the acceptor plant itself—encompassing the activity of antioxidant enzymes and phenylpropanoid pathways [82,86]. These differences are of considerable practical significance in the context of agroforestry system design and species selection in organic farming, where the controlled allelopathic potential of trees may be deliberately exploited for biological weed suppression, whilst minimising adverse effects on crop plants [22].

From an applied perspective, the physiological effects of angiosperm tree allelochemicals provide a basis for considering their potential use in sustainable agriculture and ecological weed management. Compounds such as juglone, phenolic acids, flavonoids, and other secondary metabolites exhibit inhibitory activity against key physiological processes of competing plants, including germination, root development, photosynthesis, and mineral acquisition [22,23,29,78]. This creates opportunities for the development of natural herbicidal products and for the integration of allelopathic tree species into agroforestry systems designed to suppress undesirable vegetation.

Tree-derived litter and plant residues may also contribute to biological weed control by modifying rhizospheric conditions and releasing bioactive compounds during decomposition [14,17,87]. However, practical implementation requires a mechanistic understanding of allelochemical persistence, soil transformation, and species-specific sensitivity. The same compounds that suppress weeds may also negatively affect desirable crops or interfere with soil ecological processes. Therefore, future applications should focus on targeted exploitation of allelopathic potential through careful selection of tree species, management of residue inputs, and development of selective strategies that maximise weed suppression while minimising adverse effects on crop productivity and soil functioning.

Taken together, the evidence reviewed herein indicates that the allelopathy of angiosperm trees is a phenomenon considerably more complex and multidimensional than would be apparent from the majority of available publications focusing on individual aspects of phytotoxicity. Integration of knowledge concerning the multi-level physiological effects of allelochemicals with data on their environmental chemistry, rhizospheric transformation, and interactions with the soil microbiome is an indispensable step towards a comprehensive understanding of the ecological and applied significance of angiosperm tree allelopathy [2,16,33].

## 5. Conclusions

**Figure 9:**
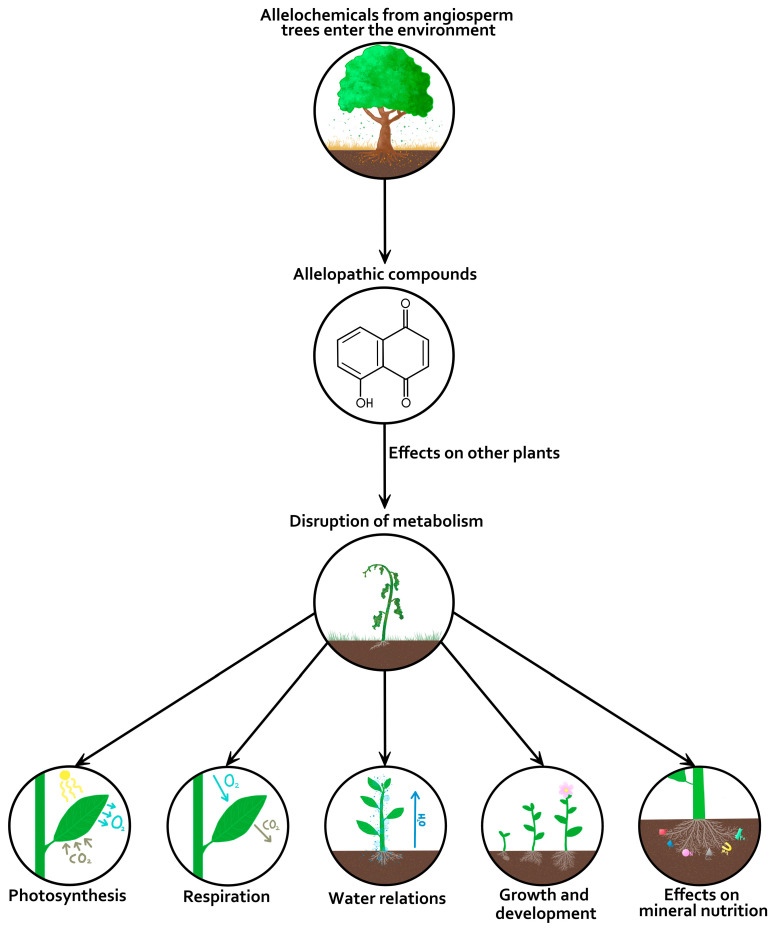
Conceptual summary of the reported effects of angiosperm tree-derived allelochemicals on acceptor plants, based on evidence synthesised in this systematic review. Allelochemicals released by mature angiosperm trees into the surrounding environment can affect acceptor plants across multiple stages of development, including germination, seedling establishment, and subsequent growth. Exposure to these compounds is associated with reduced germination success and impaired early seedling vigour, followed by growth inhibition and developmental abnormalities in later ontogenetic stages. These effects are mediated through cumulative physiological disturbances, including disruption of key metabolic processes, impaired resource acquisition, and reduced biomass accumulation. Collectively, these alterations lead to weakened plant performance and, in severe cases, failure of establishment and increased mortality. The figure presents a conceptual model integrating the principal mechanisms consistently reported across studies included in this systematic review.

The present review demonstrates that allelochemicals emitted by angiosperm trees exert a multi-level and highly integrated influence on the functioning of acceptor plants. These interactions encompass concurrent disruption of photosynthesis, water relations, mineral nutrition, respiration, and growth, resulting in systemic destabilisation of plant metabolism (Figure 9).

The most sensitive elements of the plant physiological response are processes associated with electron transport in mitochondria and chloroplasts, antioxidant enzyme activity, and cell membrane integrity. These disturbances give rise to secondary effects encompassing elevated reactive-oxygen-species levels, lipid peroxidation, and reduced cellular energy efficiency.

An important mechanism of allelochemical action is also the effect on water and mineral relations, encompassing disruption of stomatal functioning, imbalance of the chemical potential of water within cells, restricted mineral nutrient uptake, and alterations in root system architecture. As a consequence, water and ion transport are restricted, further amplifying metabolic stress.

The findings also indicate that allelopathic interactions are concentration- and exposure time-dependent, and their consequences may be both short-term—manifested as physiological disturbances—and long-term, resulting in structural alterations and persistent impairment of plant functioning.

In conclusion, the allelopathy of angiosperm trees constitutes a significant ecological factor regulating plant functioning in ecosystems, acting through complex and mutually interconnected physiological, biochemical, and environmental mechanisms. The findings presented herein underscore the need for further research into the specific action pathways of individual allelochemical groups and their interactions with environmental factors. Future investigations should place particular emphasis on the identification and characterisation of specific allelochemical groups responsible for the observed physiological effects, including their soil metabolism and interactions with the rhizospheric microbiome. Elucidation of the molecular mechanisms underlying interspecific and inter-organ differences in sensitivity—with particular attention to root system responses and hormonal regulation—will also be of considerable importance. Multi-omics approaches—encompassing transcriptomics, metabolomics, and proteomics—should assume an increasingly prominent role, enabling integration of plant responses at both the cellular and whole-organism levels.

From an applied perspective, allelopathy may provide the basis for the development of natural plant-protection strategies, including bioherbicides and weed-suppression methods in agricultural and agroecological systems. Compounds of arboreal origin may serve as alternatives to synthetic herbicides, particularly in the context of mounting pressure to reduce the use of chemical plant-protection products. Concurrently, potential adverse effects—such as autotoxicity and long-term alterations in soil properties—must be taken into account, as these may compromise the stability of agricultural ecosystems. Integration of allelopathic knowledge into agricultural practice may, in future, contribute to the development of more sustainable crop-production systems.

## Data Availability

No new data were created or analysed in this study. Data sharing is not applicable to this article.

## Abbreviations

The following abbreviations are used in this manuscript:

APX	ascorbate peroxidase
ATP	adenosine triphosphate
CAT	catalase
Ci	intercellular CO_2_ concentration
C:N	carbon-to-nitrogen ratio
CO_2_	carbon dioxide
Fe(III)	ferric iron ion
Gs	stomatal conductance
H^+^-ATPase	membrane-bound proton ATPase
IAS	International Allelopathy Society
IAA oxidase	indole-3-acetic acid oxidase
MDA	malondialdehyde
NADPH	nicotinamide adenine dinucleotide phosphate (reduced form)
NH_4_^+^	ammonium ion
NO_3_^−^	nitrate ion
Pn	net photosynthesis
PAL	phenylalanine ammonia-lyase
POD	peroxidase
PRISMA	Preferred Reporting Items for Systematic Reviews and Meta-Analyses extension
PSI	photosystem I
PSII	photosystem II
ROS	reactive oxygen species
SOD	superoxide dismutase
Tr	transpiration rate
VOCs	volatile organic compounds
